# Influence of Microbiota on Diabetic Foot Wound in Comparison with Adjacent Normal Skin Based on the Clinical Features

**DOI:** 10.1155/2019/7459236

**Published:** 2019-08-19

**Authors:** Ji-Ung Park, Bumjo Oh, Jung Pyo Lee, Min-Ha Choi, Min-Jung Lee, Bong-Soo Kim

**Affiliations:** ^1^Department of Plastic and Reconstructive Surgery, Seoul National University Boramae Medical Center, Seoul 07061, Republic of Korea; ^2^Department of Family Medicine, Seoul National University Boramae Medical Center, Seoul 07061, Republic of Korea; ^3^Department of Internal Medicine, Seoul National University Boramae Medical Center, Seoul 07061, Republic of Korea; ^4^Department of Life Science, Hallym University, Chuncheon, Gangwon-do 24252, Republic of Korea; ^5^Multidisciplinary Genome Institute, Hallym University, Chuncheon, Gangwon-do 24252, Republic of Korea

## Abstract

Diabetic foot ulcer (DFU) is a complication experienced by diabetic patients and does not heal well in an altered wound environment. Although diverse microbes in DFU were detected, little is known about their influences on diabetic foot wound (DFW) and the association with the skin microbiota in normal tissue from the same patients according to clinical features. We aimed to analyze the microbiota in normal skin and DFW tissue from the same subject and predict their roles based on clinical features. We analyzed the microbiota in normal skin and DFW tissue from the same subject and compared the associated members of microbiota with clinical parameters. The diversity of skin microbiota was higher than that of DFW tissues, along with compositional differences. In addition, different microbes were associated with clinical features. The proportions of Bacteroidetes,* Prevotella*,* Peptoniphilus*,* Porphyromonas*, and* Dialister* were higher in the severe groups than of the mild groups, whereas that of Firmicutes was lower in the severe groups. According to wound severity, the microbiota could be related to inflammation, damaging host cell membrane, and pathogenicity through lipopolysaccharide biosynthesis, cellular antigens, and protein digestion metabolism. The predicted DFW microbiota functions according to systemic diabetic status defined by ESRD and HbA1c, differed from those presented by wound severity. Results indicate that the microbiota in normal skin is related to the colonizing microbes in DFW tissue according to clinical features and the different microbes can play important roles in DFW prognosis. This information can be applied to prevent and manage DFW by modulating the microbiota.

## 1. Introduction

The prevalence of diabetes has increased and was predicted to affect 629 million patients by 2045, resulting in socioeconomic problems related to poor management [1]. Seventy percentage of lower limb amputations are developed in diabetic foot patients [2–4] and 50% patients undergo amputation of the contralateral lower limb within 5 years. Fifty to seventy percentage of patients with diabetic limb amputation die within 5 years [5–7]. Infections in diabetic foot lesions do not heal well because of factors such as an impaired vascular supply, increased inflammation, metabolic abnormalities, neuroses, and edema [8]. Additionally, the foot has a specific anatomical structure that allows an infection to reach other areas [9, 10]. Therefore, accurate analysis of wound infection is essential for improving the prognosis of diabetic foot as a problematic chronic infectious wound.

Diverse bacteria, including* Staphylococcus* and* Streptococcus*, and fungi are frequently detected in clinical wounds based on the severity of diabetic foot according to the perfusion, extent, depth, infection and sensation (PEDIS) classification [11]. Although various antibiotic regimens have been used to treat diabetic foot infections, they are insufficient for treating infected wounds. This may be because of complex interactions such as quorum sensing communication among various microbes in the wounds. Moreover, ischemic condition such as arteriosclerosis obliterans, multiple stenosis of peripheral lower extremity vessels in diabetic foot wound interfere with the effect of antibiotics therapy. Recent studies reported that chronic wounds contain polymicrobial biofilm exceeding the identification capabilities of culture methods [12, 13]. In particular, deep tissue cannot be easily harvested by conventional swab culture protocol, and diagnosis frequently fails for deep infections [14, 15]. Diabetic foot wounds (DFWs) are exposed to skin commensal bacteria that can colonize the wound as multilayered microbiota surrounded by a self-produced protective extracellular biofilm [16]. Several studies have shown higher microbial diversity in diabetic foot ulcers (DFUs) by high-throughput sequencing based on the 16S rRNA gene than previous culture-based assay [17–20]. However, there is little information about skin microbiota in normal tissue from patients with DFW and comparison with microbiota in DFW tissues according to clinical features.

The aim of this study was to analyze the differences in microbiota between normal skin and DFW from the same subject using high-throughput sequencing based on 16S rRNA genes and determine whether any members are associated with clinical parameters such as severity, infection depth, and etiology. In addition, the influence of microbiota in DFW prognosis was analyzed by predicted microbiota function. These results can be applied in the clinical treatment and prediction of DFW in future.

## 2. Materials and Methods

### 2.1. Ethical Approval

This study was approved by the Institutional Review Board of Seoul National University Boramae Medical Center (No. 26-2016-180) and performed in accordance with the Declaration of Helsinki.

### 2.2. Subjects and Sample Acquisition

Twenty patients with DFW were recruited at the Department of Plastic and Reconstructive Surgery in Seoul National University Boramae Medical Center. All participants provided informed consent, and identifying information was blinded at sampling. Clinical data acquired through interview and chart review included gender, age, severity of infection, stage of disease, and results of blood analyses including presence of end-stage renal disease (ESRD), hemoglobin A1c (HbA1c), and serum creatinine levels.

Specimens were collected from normal skin and DFW tissue of each subject. A metzenbaum scissor or a blade was used to debride the necrotic tissue including unhealthy granulation tissue of each wound. The acquired wound tissues were placed in sterile tubes and stored at -80°C until DNA extraction. The normal skin sample of the ipsilateral ankle in the same patient was obtained from the mid-dorsum of the ankle surface using sterile cotton swabs (EASY SWAB, Hanil-Komed Inc., Seongnam, Gyeonggi-do, South Korea) and frozen at -80°C until DNA extraction. DNA contamination of the reagents was evaluated using negative controls consisting of unused swabs.

### 2.3. DNA Extraction and MiSeq Sequencing

Metagenomic DNA was extracted from 40 samples (20 tissues and 20 skin swabs) using an RNeasy PowerMicrobiome kit (Qiagen, Hilden, Germany). The extracted DNA was purified using the DNeasy PowerClean Pro clean-up kit (Qiagen) and confirmed by 1% agarose gel electrophoresis. DNA was not extracted from one skin swab sample, and hence it was excluded from further experiments. Samples were prepared for high-throughput sequencing as described previously [21]. The V1–V3 regions of the 16S rRNA gene were amplified using primers with an adapter (forward: 5′-adapter [TCGTCGGCAGCGTCAGATGTGTATAAGAGACAG]-GAGTTTGATCMTGGCTCAG-3′; reverse: 5′-adapter [GTCTCGTGGGCTCGGAGATGTGTATAAGAGACAG]-ATTACCGCGGCTGCTGG-3′) from extracted DNA using a C1000 Touch thermal cycler (Bio-Rad, Hercules, CA, USA). The amplification was followed by the protocol for preparing a 16S metagenomic sequencing library for the MiSeq system (Illumina, Inc., San Diego, CA, USA). Negative controls (blank swab) were also included in both steps of DNA extraction and amplification and evaluated by gel electrophoresis after each step. The purification and size selection were performed using the Agencourt AMPure XP beads (Beckman Coulter, Brea, CA, USA) from amplified products. Then, index PCR was performed using the Nextera XT index kit (Illumina, Inc.), and the purification and size selection were performed again using Agencourt AMPure XP beads (Beckman Coulter). The purified products were quantified using a PCR Thermal Cycler Dice Real Time System III (TaKaRa, Shiga, Japan). Equimolar concentrations of each library from the samples were pooled and sequenced using the Illumina MiSeq system (300-bp paired ends) according to the manufacturer's instructions.

### 2.4. Data Analysis

The obtained sequences were processed using the CLC genomic workbench (ver. 8.5.1) with the Microbial Genomics Module (Qiagen). Raw sequences of short-read lengths (< 200 bp/pair) and low-quality scores (Q < 25) were removed, and paired reads were merged with a mismatch score of overlap sequences. Primer sequences were removed from the merged sequences, and short-read lengths were removed (< 430 bp of merged reads). Chimeric sequences were removed using the UPARSE tool [22]. Then, sequences were clustered to operational taxonomic units (OTUs) using the 97% sequence similarity based on the EzTaxon-e reference database [23]. The representative sequence in each OTU cluster was identified for taxonomically by the EzTaxon-e database. To compare the diversity indices among samples, the number of reads in each sample was normalized by random subsampling, and calculated using the MOTHUR program [24]. Principal coordinate analysis (PCoA) plots were obtained to compare microbiota among samples using Calypso [25]. The functional prediction of the microbiota was performed using the PICRUSt (ver. 1.0.0) [26].

### 2.5. Statistical Analysis

The differences of microbiota between samples were analyzed using the Mann-Whitney U-test and Kruskal-Wallis test in R software. Permutation tests were used to calculated statistical differences in microbiota in PCoA. Significantly different predicted Kyoto Encyclopaedia of Genes and Genomes (KEGG) pathways were determined using the Kruskal-Wallis H-test, and a post hoc test was performed using the Tukey-Kramer method [27]. Results with p value < 0.05 were considered as statistically significant.

## 3. Results

### 3.1. Clinical Characteristics of the Subjects

**Figure 1:**
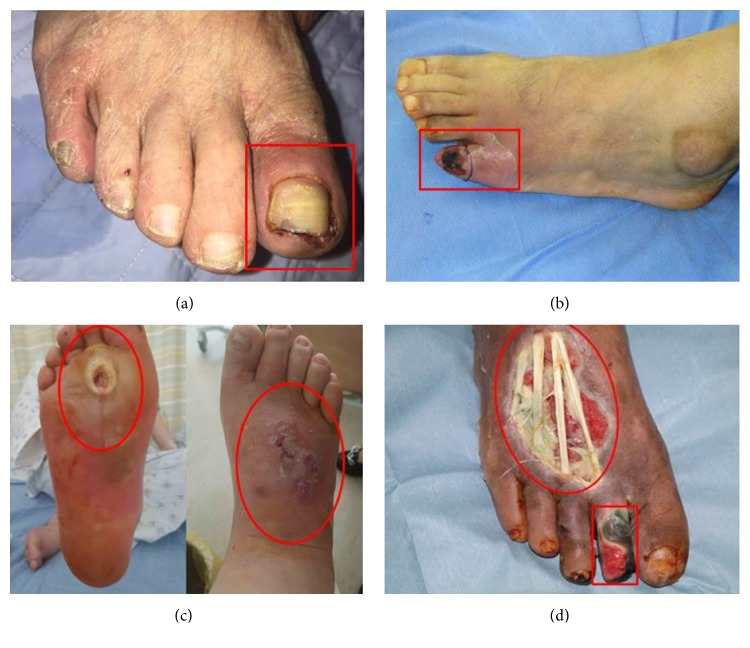
Clinical photographs according to the severity of Wagner classification. (a) Grade 1 superficial ulcer of left big toe (ischemic type), (b) grade 2 deep ulcer of left fifth toe (ischemic type), (c) grade 3 perforating ulcer from plantar surface to dorsum of foot with abscess and osteomyelitis (neuropathic type), and (d) grade 4 local gangrene of right 2^nd^ toe and dorsum of foot (mixed ischemic and neuropathic type).

Overall clinical features and categorization criteria are summarized in Table S1. Twenty subjects were enrolled with a mean age of 62.45 ± 12.46 years, and sixteen subjects were male. Patients were categorized into two subgroups depending on several clinical factors believed to affect wound infection. First, we categorized the patients according to the cause. There were 12 diabetic foot wounds with dominantly ischemic cause and 8 with neuropathic cause. In terms of the Wagner classification scoring system, 5 patients wounds were classified as mild (≤ 2), with no bony involvement, while 15 were considered severe (> 3) (Figure 1). Infection severity was evaluated by PEDIS classification and divided as 5 patients with mild infection and 15 with severe infection. There were 9 patients with ESRD who received dialysis treatment. Poor diabetes controlled patients with more than 8% HbA1c were five among 14 available patients. Severe arterial occlusion of lower extremity was observed in ten subjects among 19 patients.

### 3.2. Comparison of Microbiota between Skin and Tissue of Subjects

**Figure 2:**
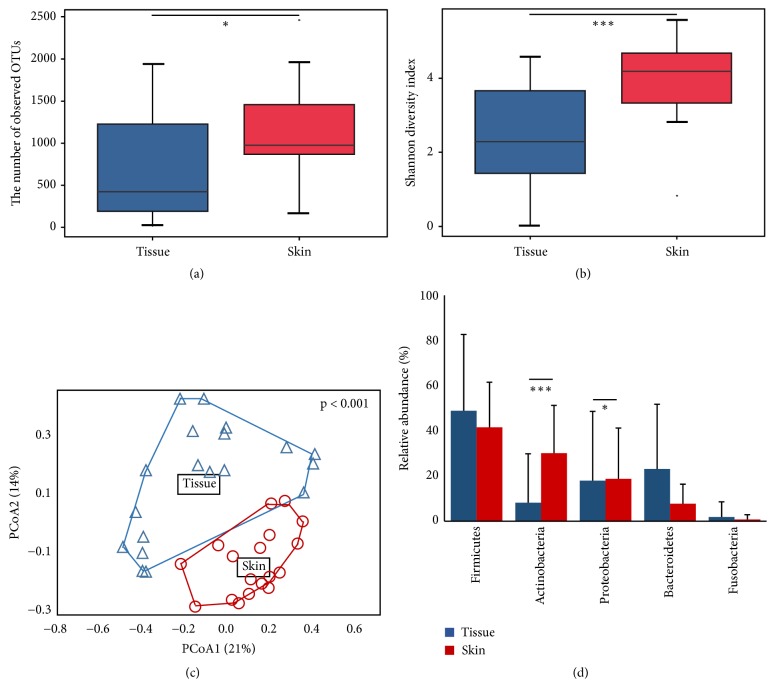
Comparison of microbiota between normal foot skin and diabetic foot wound tissue. (a) Number of observed OTUs and (b) Shannon diversity index of microbiota were compared using boxplot. Median values and lower/upper quartiles were shown in boxplot. (c) Difference in microbiota between skin and tissue samples was analyzed in PCoA plot based on Bray-Curtis distance. P value in PCoA plot was calculated by permutation test. (d) Composition of phylum was compared between groups. Mean values and standard deviations were shown in bar-chart. The significance of differences between groups was calculated by Mann-Whitney U-test (*∗*p < 0.05, ∗∗∗p < 0.001).

A total of 4,169,039 reads (2,161,104 from 20 tissue samples and 2,007,935 from 19 skin samples) were analyzed after trimming (Table S2). The read number in each sample was normalized to 37,000 by random subsampling. The Good's coverage was over 97% in all samples. The microbiota was compared between the tissue and skin swab samples (Figure 2). The number of observed OTUs and diversity index were higher in the skin than in the tissue (p < 0.05). The PCoA plot showed that the microbiota in the tissue was different from that in the skin (Figure 2(c)) (p < 0.001). The composition of phylum in tissues was compared to that in skin samples (Figure 2(d)). Six phyla were detected in tissue samples and 22 in skin samples. Firmicutes, Actinobacteria, Proteobacteria, Bacteroidetes, and Fusobacteria were dominant phyla (> 99% in all microbiota) in both tissue and skin samples. The relative abundances of Actinobacteria (30.14%) and Proteobacteria (18.78%) were significantly higher in skin samples than those (8.14% and 17.95%) in the tissue samples, respectively (p < 0.05).

The differences in microbiota between tissue and skin samples were detailed at the genus level. Sixty-nine genera were detected in tissue samples and 390 genera in skin samples. The relative abundances of frequently detected genera (> 1% of total microbiota in each sample type) were compared between the tissue and skin swab samples (Figure S1). The relative abundances of* Pseudomonas*,* Bacteroides*, and* Enterococcus* were significantly higher in tissue samples than in skin samples (p < 0.05), whereas those of* Staphylococcus*,* Corynebacterium*,* Streptococcus*,* Propionibacterium*,* Methylobacterium*,* Lactobacillus*,* Rothia*,* Sphingomonas*,* Acinetobacter*,* Brevibacterium*,* Micrococcus*, and* Paracoccus* were higher in skin samples than in tissue samples (p < 0.01).

### 3.3. Different Microbes according to Clinical Features

**Figure 3:**
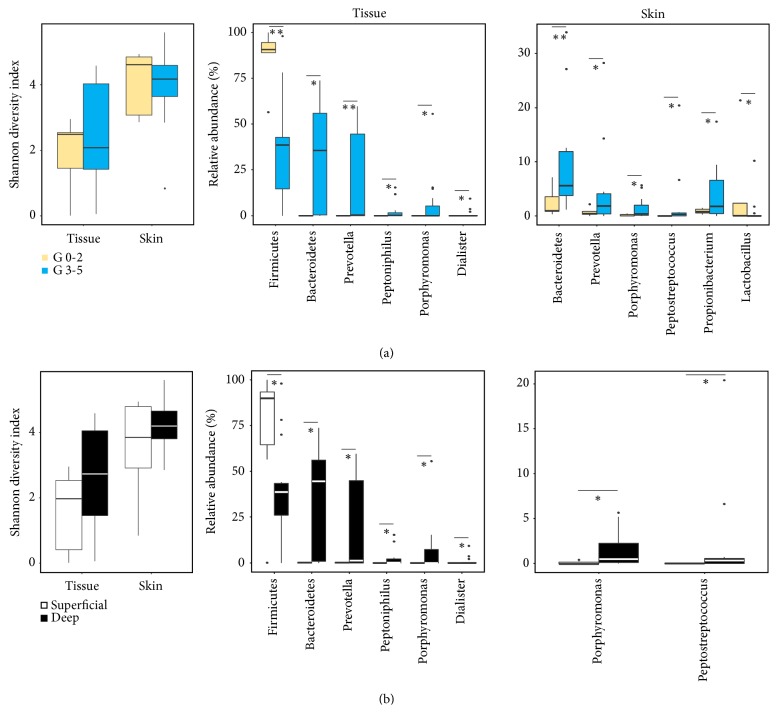
Diversity and significantly different microbial taxa in skin and tissue samples were compared between mild and severe groups. Diversity and significantly different members of microbiota were compared between (a) lower grade (G0-2) and higher grade (G3-5) of Wagner classification and (b) superficial infection and deep infection. Median values and lower/upper quartiles were shown in boxplot. The significance of differences between groups was calculated by Mann-Whitney U-test (∗p < 0.05, ∗∗p < 0.01).

The microbiota in tissue samples was different to that in skin samples. Thus, we compared the microbiota according to clinical factor groups in each sample type. The higher number of significantly different microbial members was detected by Wagner classification and wound depth (Figure 3). According to the Wagner classification groups, the diversity index of microbiota from both tissue and skin samples was higher in grade 0–2 (G0-2) group than in grade 3–5 (G3-5) group (Figure 3(a)). However, the difference in diversity index was not statistically significant (p > 0.05). Firmicutes in tissue samples was more abundant in G0-2 group than in G3-5 group (p < 0.01), and* Lactobacillus* in skin samples was higher in G0-2 group (p < 0.05). The higher abundant microbial members in G3-5 group were similar between tissue and skin samples. Bacteroidetes,* Prevotella*,* Peptoniphilus*,* Porphyromonas*, and* Dialister* in tissue samples were higher in G3-5 group than in G0-2 group (p < 0.05), and Bacteroidetes,* Prevotella*,* Porphyromonas*,* Peptostreptococcus*, and* Propionibacterium* in skin samples were higher in G3-5 group (p < 0.05). Different microbial members between superficial and deep wound groups were similar to those in previous Wagner classification group (Figure 3(b)). The proportion of Firmicutes in tissue samples was higher in superficial wound than in deep wound samples, whereas Bacteroidetes,* Prevotella*,* Peptoniphilus*,* Porphyromonas*, and* Dialister* in tissue samples and* Porphyromonas* and* Peptostreptococcus* in skin samples were higher in deep wound samples (p < 0.05).

Similar microbial members differed among groups of other clinical factors (Figures S2 and S3). For ESRD factor, the proportions of Bacteroidetes,* Prevotella*,* Peptoniphilus*, and* Porphyromonas* in tissue samples and* Finegoldia* in skin samples were higher in the group without ESRD than in the group with ESRD, whereas* Lactobacillus* in skin samples was higher in the group with ESRD (p < 0.05). For HbA1c, the proportions of Bacteroidetes,* Peptoniphilus*, and* Streptococcus* in tissue samples and* Peptoniphilus* and* Streptococcus* in skin samples were higher in > 8% group than in < 8% group (p < 0.05). For severity, the proportion of Firmicutes in tissue samples was higher in the mild group than in the severe group, whereas Bacteroidetes in tissue samples and Bacteroidetes and* Peptoniphilus* in skin samples were higher in severe group (p < 0.05). For the causal factor, the proportion of* Anaerococcus* in skin samples was higher in the neuropathic group than in the ischemic group (p < 0.05).

### 3.4. Predicted Functions of Microbiota according to Clinical Features

**Figure 4:**
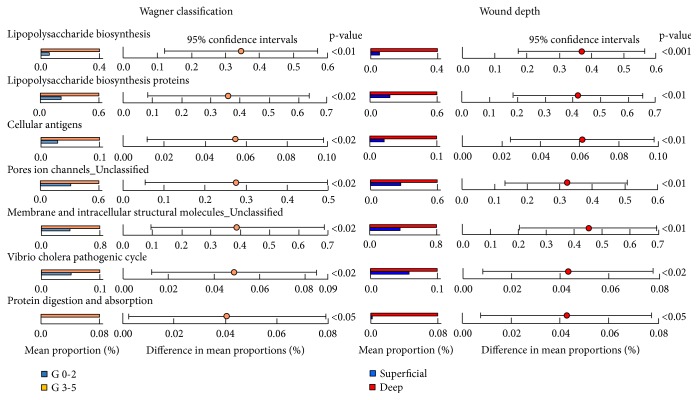
Predicted functions of microbiota in DFW tissue according to Wagner classification and wound depth were compared in post hoc plots. Seven pathways were predicted to be commonly highly represented in higher grade (G3-5) of Wagner classification and deep infection. Significantly different predicted pathways were determined using the Kruskal-Wallis H-test, and a post hoc test was performed using the Tukey-Kramer method.

Different microbes were detected according to clinical factor groups and sample types. We compared the predicted functions of microbiota between skin and tissue samples according to clinical factors. Eighty KEGG orthologues (KO) were predicted significantly different between microbiota of skin and tissue samples (p < 0.05) (Table S3). Twenty-two metabolisms of microbiota were higher in tissue than skin samples, whereas 58 metabolisms were higher in the skin than tissue samples. Then, we compared significantly different pathways of DFW tissue microbiota between clinical groups. Eleven pathways were predicted commonly different between groups of Wagner classification and wound depth (p < 0.05). Seven pathways (lipopolysaccharide biosynthesis, lipopolysaccharide biosynthesis proteins, cellular antigens, pores ion channels, membrane and intracellular structural molecules,* Vibrio cholera* pathogenic cycle, and protein digestion and absorption) were predicted highly represented in severe group than mild group (p < 0.05) (Figure 4), whereas four pathways (ABC transporters, phosphotransferase system, glycolysis/gluconeogenesis, and ascorbate and aldarate metabolism) were highly represented in mild group than severe group (p < 0.05) (Figure S4). The proportions of five pathways were predicted higher in the group without ESRD than in the group with ESRD (p < 0.05) (Table S4). The proportions of seventeen pathways were predicted to be higher in HbA1c > 8% group than < 8% group, whereas those of eleven pathways were predicted lower in HbA1c > 8% group (p < 0.05) (Table S5).

## 4. Discussion

The microbiota of DFW tissue was analyzed and compared to that of normal foot skin in the same patients. Although the same phyla were dominant in both wound tissue and skin samples, more diverse microbes were detected in the skin samples. Significantly different microbial members were detected according to clinical factors. Similar members were detected in both wound tissue and skin sample even in different clinical factor groups. Predicted functions of microbiota were also significantly different among clinical groups, and similar pathways were detected in the DFW tissue of severe groups. Particularly, the proportions of pathways related to inflammation and tissues damage, such as lipopolysaccharide (LPS) biosynthesis, cellular antigens, and protein digestion, were higher in severe groups than mild groups. These results indicate that the microbiota of skin and tissue can be associated with the progress or severity of DFW.

The microbiota in DFW tissue differed from that in normal skin obtained from the same patients. The diversity was lower in DFW tissue than normal foot skin (Figure 2), since the DFW tissue exhibits a specific microenvironment including alterations in sweat glands, sebaceous glands, or hair follicles. The microbiota in DFW can form by the random settlement of early colonizers, and subsequently unique biofilm develops after competition among various colonizers [28]. In addition, the sampling location can lead to differences in the microbiota in DFW tissue. The skin sample was obtained from the skin surface by swab sampling, whereas the DFW tissue was obtained using the excisional method. Thus, the unique microbiota in wound tissues including wound fluid and matrix can be detected. The difference in microbiota and lower diversity in wound tissue than skin from the same patients was consistent with a previous study [19]. Lower diversity because of the overgrowth of some potential pathogens can be trigger inflammation, or inflammation could drive down diversity by creating an inhospitable growth environment.

Although dominant members of microbiota were similar in both samples, the diversity and microbiota composition were different between skin and tissue (Figures 2(d) and S1). The higher abundances of Actinobacteria,* Staphylococcus*,* Corynebacterium*, and* Propionibacterium* in skin samples than tissue samples (p < 0.01) were consistent with previous result [29]. These microbes benefit the host such as regulating the host's adaptive immune response or inhibiting the growth of pathogenic organisms. However, they can also produce or participate in chronic infections under certain conditions [30]. The relative abundances of anaerobes (*Bacteroides* and* Enterococcus*) and* Pseudomonas* were higher in tissue samples (p < 0.05) as previously reported [31, 32].* Corynebacterium* is localized in the upper regions, where the oxygen content is relatively high, while anaerobes and* Pseudomonas* are located in deeper in the wound bed [31, 33]. More number of pathways (58 pathways among 80 KO) were predicted significantly higher in the microbiota of skin than tissue samples (Table S3). This could be because of the higher diversity of microbiota in the skin than tissue samples and the unique microenvironment of DFW tissue comparing to normal skin. These results revealed microenvironment-based differences in the microbiota between skin and tissue samples.

The microbes that differed significantly according to clinical characterization are summarized in Table 1. The higher abundance of Firmicutes in DFW tissue was detected in mildly severe, superficial infection, and low-grade Wagner classification. However, the higher abundances of Bacteroidetes,* Prevotella*,* Peptoniphilus*,* Porphyromonas*, and* Dialister* in DFW tissues were detected in more severe, deep infection, and high-grade Wagner classification. The higher abundance of Firmicutes in relatively low-grade and mild DFW tissue was consistent with a previous study [34]. A longitudinal shift of wound microbiota occurred from Firmicutes to Proteobacteria, which resulted in a corresponding decline in wound healing. Our results showed that anaerobic bacteria were significantly higher in DFW of the severe group including deep infection. The correlation between abundance of anaerobes and ulcer depth was reported [17]. Anaerobes in the wound can impair wound healing and increase the severity of wounds through their virulence factors such as adhesion factors, tissue-damaging exoenzymes, and antiphagocytic factors [35]. The pathogenic effects of anaerobes can be increased by interaction with aerobes, which can cause polymicrobial infection of DFW. Aerobes consume oxygen, inducing tissue hypoxia, and facilitate growth of anaerobes by reducing the redox potential, causing impairment of host immune cell function [36]. Notably, the same anaerobes in the normal foot skin site were also abundant in the same severe subjects. This is because of the survival capability of anaerobes including* Prevotella*,* Porphyromonas*, and* Bacteroides* in the presence of air [37, 38]. This result suggests that the indigenous microbiota in the skin can transfer to wound tissue and increase the severity and impairment of the wound. Skin microbiota is influenced by lifestyle, environments, and other individuals; however, it remains stable over time in the same individual [39, 40]. Therefore, the indigenous skin microbiota can transfer to wound tissue in the same individual. Although further studies are necessary to validate the severity of associated microbes, the presence of these microbes in normal foot skin can predict the progress or develop preventive strategies by targeting them. The higher abundance of* Anaerococcus* was detected only in the skin sample of neuropathic diabetic foot patients. No differences were found in members of DFW tissue between ischemic and neuropathic patients [20].

The influences of different microbiota on DFW tissue were predicted by significantly different microbiota pathways (p < 0.05). Seven pathways were predicted commonly highly represented in severe group than mild group according to Wagner classification and wound depth (Figure 4). LPS is a bacterial cell wall component that can induce the immune response of macrophages to increase nitric oxide (NO) production. The high amount of NO can be cytotoxic and damage the surrounding tissue resulting in increased inflammation [41]. Bacterial antigens can be a virulence factor such as superantigens and damage membranes of host cells leading to cell lysis [42]. Bacterial ion channels are components of signal transduction pathways and employed to handle environmental challenges [43]. The higher abundance of pore ion channels in the severe group can be an adaptive response of the microbes in the DFW tissue of this group. The higher abundance of* Vibrio cholera* pathogenic cycle in the severe group than mild group can be associated with higher pathogenicity or virulence of microbes in the severe group. Protein digestion and absorption can be associated with tissue damage by microbes. In contrast, general metabolisms such as ABC transporters, the phosphotransferase system, glycolysis/glyconeogenesis, and ascorbate/aldarate metabolism were predicted to be higher in the mild group than in the severe group (Figure S4). These differences could be because of the different microbiota between mild and severe group. These results indicate that microbes in the severe group can be related to destruction of the tissue membrane.

The higher abundances of Bacteroidetes,* Prevotella*,* Peptoniphilus*, and* Porphyromonas* in DFW tissue were detected in the group without ESRD, and Bacteroidetes,* Peptoniphilus*, and* Streptococcus* were predominant in the > 8% HbA1c group. Both ESRD and HbA1c are associated with blood sugar level, diabetes management, and systemic status. Similar microbes were detected higher in tissue with poor status, which were similar to those in the severe group. Therefore, patients with poorly controlled diabetes could progress to severe status by microbiota. Although higher abundant microbes in the poor-status ESRD and HbA1c group were similar to microbes in severe groups, the predicted functions of microbiota were different to those of severe groups (Tables S4 and S5). In particular, pathways related to LPS and protein digestion did not detect significantly in the poor status of ESRD and HbA1c. This indicated that different microbiota according to ESRD and HbA1c could play different roles in DFW tissue. Systemic status of diabetes management such as blood sugar level, chronic renal function, and presence of varicose vein could influence on microbiota in DFW tissue. Further studies are necessary to validate the relationship between diabetic management or systemic status and the role of microbiota in DFW tissue.

A diabetic foot ulcer is a concerning public health problem, and appropriate prevention and effective early treatment are required to avoid catastrophic amputation surgery [44, 45]. However, chronic infection makes initial treatment of diabetic foot difficult and carries a risk of systemic deterioration because the foot infection can easily spread following the foot compartment system. Diabetic foot infection is underestimated because of inexactitude of standard culture techniques although that includes complex mixed polymicrobial communities with high concentrations [8, 17, 46]. Notably, the susceptibility to infections can be altered according to various clinical conditions of patients, along with the microbial composition. Accurate identification helps to prudent use of antibiotics without abuse them. Therefore, the analyses using high-throughput sequencing in the present study are relevant to understanding the polymicrobial biofilm in DFW tissue.

The limitations of this study are the relatively small number of diabetic foot patients studied and the single time point sampling. However, the microbiota of DFW tissue was compared with that of normal foot skin from the same subjects, and different microbes with their predicted functions were detected according to clinical features. In particular, we found that the skin microbiota could transfer to DFW tissue and the association of these microbes with the severity and poor systemic diabetic status. This information can be applied to modulate the skin microbiota of diabetes patients to reduce the severity of diabetic foot infection. Further studies with large sample size, and host-microbiome interactions with host genetic, immunologic, and metabolic factors are needed to validate obtained results and to develop novel management of diabetic foot patients.

## 5. Conclusions

We compared the microbiota in normal foot skin and DFW tissue from the same subject for identifying the different microbes associated with clinical features and predicting their functions in wound. The diversity of microbiota was lower in DFW tissue than in normal skin because of the unique microenvironment such as the oxygen concentration and wound tissue structure. Significantly different microbes according to severity features were similar in Wagner classification and wound depth. The abundant microbes in the severe group could be related to inflammation, damage of host cell membrane, virulence of microbes via LPS biosynthesis, bacterial antigens, the pathogenic cycle of pathogens, and protein digestion metabolism in predicted function analysis. In addition, the significantly different microbes were identified according to the systemic status of diabetic management such as ESRD and HbA1c. The predicted functions of these microbes in DFW tissue were different to severe groups because of their different interactions with the host through systemic diabetic status. These microbes were also detected in normal skin. These results indicated that the skin microbiota can transfer to wound, then it can play roles in DFW prognosis. Therefore, the identification of normal skin microbiota can be used to early prediction of the DFW prognosis, and the modulation of skin microbiome can apply to prevent or manage DFW in patients with diabetes. Although further studies are necessary to validate these findings, obtained results can help to understand the association of normal skin microbiota with DFW tissue microbiota and to develop novel management of diabetic foot patients.

## Figures and Tables

**Table 1:** Significantly different microbes according to clinical groups.

Clinical	Significantly abundant microbes			
characterization	Tissue	Skin	Tissue	Skin
Wagner classification	G 0-2		G3-5	
	Firmicutes	*Lactobacillus*	Bacteroidetes	Bacteroidetes
			*Prevotella*	*Prevotella*
			*Peptoniphilus*	*Peptostreptococcus*
			*Porphyromonas*	*Porphyromonas*
			*Dialister*	*Propionibacterium*

Infection type	Superficial		Deep	
	Firmicutes		Bacteroidetes	*Porphyromonas*
			*Prevotella*	*Peptostreptococcus*
			*Peptoniphilus*	
			*Porphyromonas*	
			*Dialister*	

Severity	Mild		Severe	
	Firmicutes		Bacteroidetes	Bacteroidetes
				*Peptoniphilus*

ESRD	Yes		No	
		*Finegoldia*	Bacteroidetes	*Lactobacillus*
			*Prevotella*	
			*Peptoniphilus*	
			*Porphyromonas*	

HbA1c	< 8%		>8%	
			Bacteroidetes	
			*Peptoniphilus*	*Peptoniphilus*
			*Streptococcus*	*Streptococcus*

Etiology	Ischemic		Neuropathic	
				*Anaerococcus*

## Data Availability

The sequencing reads obtained from this study are available in the EMBL SRA database under study accession number PRJEB30094 (http://www.ebi.ac.uk/ena/data/view/PRJEB30094).

## Supplementary Materials

Supplementary Materials Table S1: clinical characteristics of subjects. Table S2: summary of diversity indices after normalized reads obtained from this study. Table S3: significantly different KEGG pathways of microbiota between normal skin and diabetic foot wound (DFW) tissue. Table S4: significantly different KEGG pathways of microbiota in diabetic foot wound (DFW) tissue between group with ESRD and group without ESRD. Table S5: significantly different KEGG pathway of microbiota in diabetic foot wound (DFW) tissue between HbA1c < 8% and > 8% groups. Figure S1: comparison of microbiota between normal foot skin and diabetic foot wound (DFW) tissue at genus level. Figure S2: significantly different microbial taxa in skin and tissue samples were compared between clinical groups of ESRD and HbA1c. Figure S3: significantly different microbial taxa in skin and tissue samples were compared between clinical groups of different severity and etiology. Figure S4: predicted functions of microbiota in DFW tissue according to Wagner classification and wound depth were compared in post hoc plots.
