# Supplementary material for: Influence of Microbiota on Diabetic Foot Wound in Comparison with Adjacent Normal Skin Based on the Clinical Features
Source: Biomed Res Int. 2019 Aug 19;2019:7459236. doi: 10.1155/2019/7459236 (PMC6720033; doi:10.1155/2019/7459236)
Supplement: Supplementary Materials — Table S1: clinical characteristics of subjects. Table S2: summary of diversity indices after normalized reads obtained from this study. Table S3: significantly different KEGG pathways of microbiota between normal skin and diabetic foot wound (DFW) tissue. Table S4: significantly different KEGG pathways of microbiota in diabetic foot wound (DFW) tissue between group with ESRD and group without ESRD. Table S5: significantly different KEGG pathway of microbiota in diabetic foot wound (DFW) tissue between HbA1c < 8% and > 8% groups. Figure S1: comparison of microbiota between normal foot skin and diabetic foot wound (DFW) tissue at genus level. Figure S2: significantly different microbial taxa in skin and tissue samples were compared between clinical groups of ESRD and HbA1c. Figure S3: significantly different microbial taxa in skin and tissue samples were compared between clinical groups of different severity and etiology. Figure S4: predicted functions of microbiota in DFW tissue according to Wagner classification and wound depth were compared in post hoc plots. [file 7459236.f1.docx]

**Supplementary Material**

**Table S1.** Clinical characteristics of subjects.

| **Subject** | **Sex** | **Age (years)** | **Etiology** | **Location** | **Score by Wagner classification (≤ 2, > 3)** | **Infection depth by PEDIS classification (≤ 2, > 3)** | **ESRD and dialysis** | **HbA1c (≤ 8%, > 8)** | **Cr (≤ 1.3, > .3)** | **Lower extremity - peripheral arterial occlusion** |
| --- | --- | --- | --- | --- | --- | --- | --- | --- | --- | --- |
|  |  |  |  |  |  |  |  |  |  |  |
| 1 | M | 68 | Ischemic | Left. 1st toe | 3 | 3 | N | 10.9 | 0.65 | severe occlusion |
| 2 | F | 69 | Neuropathic | Right. Heel | 2 | 1 | Y | 7.5 | 4.91 | severe occlusion |
| 3 | M | 59 | Neuropathic | Left. greatball | 3 | 3 | Y | N/A | 5.1 | severe occlusion |
| 4 | F | 79 | Neuropathic | Right. Heel | 3 | 4 | Y | 6 | 4.63 | severe occlusion |
| 5 | M | 56 | Ischemic | Left. 5th toe, ray | 3 | 3 | N | 11.2 | 1.18 | mild |
| 6 | M | 71 | Ischemic | Right. 1st toe | 1 | 2 | Y | 6.9 | 1.94 | mild |
| 7 | M | 64 | Ischemic+Neuropathic | Left. 1st, 5th toe | 4 | 4 | Y | 6.5 | 5.65 | severe occlusion |
| 8 | M | 39 | Neuropathic | Right. Malleoulus | 2 | 2 | N | 5.2 | 0.79 | no |
| 9 | F | 62 | Ischemic | Right. 1st toe | 3 | 3 | Y | N/A | 3.04 | severe occlusion |
| 10 | M | 62 | Neuropathic | Right. Sole (3rd metatarsal) | 3 | 3 | N | 11.7 | 0.79 | no |
| 11 | M | 63 | Ischemic+Neuropathic | Right. Great ball, both malleolus | 2 | 2 | Y | N/A | 1.51 | severe occlusion |
| 12 | M | 73 | Ischemic+Neuropathic | Left. 3rd toe, 5th metatarsal | 3 | 3 | N | 8.4 | 0.79 | mild |
| 13 | M | 67 | Ischemic | Left. 1st toe | 3 | 3 | N |  | 0.74 | no |
| 14 | M | 35 | Neuropathic | Right. 4th, 5th lesser ball | 3 | 3 | N | 6.8 | 0.92 | no |
| 15 | M | 55 | Neuropathic | Right. heel | 4 | 3 | Y | 7.4 | 6.01 | severe occlusion |
| 16 | M | 40 | Neuropathic | Right. 2nd, 3rd MTJ | 3 | 3 | N | N/A | N/A | N/A |
| 17 | M | 58 | Ischemic | Right. Whoe toe, Left. toe | 4 | 2 | N | 7 | N/A | mild |
| 18 | M | 73 | Ischemic | Left., Right. Multiple toe | 4 | 4 | Y | 8.6 | N/A | severe occlusion |
| 19 | F | 81 | Ischemic | Left. 3rd toe | 3 | 3 | N | N/A | N/A | severe occlusion |
| 20 | M | 75 | Ischemic | Right. Big toe | 1 | 2 | N | 6.3 | N/A | no |
| Average ± SD |  | 62.45 ± 12.46 |  |  | 2.85 ± 0.88 | 2.8 ± 0.77 |  | 7.9 ± 2.04 | 2.58 ± 2.08 |  |

**Table S2.** Summary of diversity indices after normalized reads obtained from this study.

|  |  | **Analyzed** | **Normalized** | **Observed** | **Estimated** | **Shannon** | **Good's** |
| --- | --- | --- | --- | --- | --- | --- | --- |
| **Sample** | **Subject** | **read** | **read** | **OTUs** | **OTUs (Chao1)** | **diversity index** | **coverage** |
| Tissue | 1 | 100,874 | 37,000 | 1,591 | 3,036.29 | 4.09 | 0.98 |
|  | 2 | 166,198 | 37,000 | 11 | 14.00 | 0.01 | 0.99 |
|  | 3 | 186,769 | 37,000 | 421 | 733.89 | 2.08 | 0.99 |
|  | 4 | 139,095 | 37,000 | 178 | 286.48 | 1.46 | 0.99 |
|  | 5 | 89,073 | 37,000 | 1,449 | 2,565.98 | 4.06 | 0.98 |
|  | 6 | 188,526 | 37,000 | 698 | 1,196.79 | 2.95 | 0.99 |
|  | 7 | 93,379 | 37,000 | 275 | 563.20 | 0.87 | 0.99 |
|  | 8 | 124,119 | 37,000 | 488 | 863.05 | 2.54 | 0.99 |
|  | 9 | 105,020 | 37,000 | 46 | 154.33 | 0.06 | 0.99 |
|  | 10 | 118,301 | 37,000 | 1,150 | 2,240.02 | 3.38 | 0.99 |
|  | 11 | 87,659 | 37,000 | 428 | 798.31 | 2.49 | 0.99 |
|  | 12 | 80,978 | 37,000 | 130 | 320.11 | 1.45 | 0.99 |
|  | 13 | 152,856 | 37,000 | 1,932 | 3,882.19 | 4.58 | 0.97 |
|  | 14 | 79,918 | 37,000 | 1,615 | 3,064.35 | 4.35 | 0.98 |
|  | 15 | 106,548 | 37,000 | 197 | 414.88 | 1.40 | 0.99 |
|  | 16 | 57,085 | 37,000 | 1,459 | 2,857.06 | 4.00 | 0.98 |
|  | 17 | 65,576 | 37,000 | 24 | 40.50 | 0.06 | 0.99 |
|  | 18 | 125,367 | 37,000 | 982 | 1,675.50 | 3.55 | 0.99 |
|  | 19 | 56,586 | 37,000 | 370 | 641.69 | 2.04 | 0.99 |
|  | 20 | 37,177 | 37,000 | 255 | 546.67 | 1.45 | 0.99 |
| Skin | 1 | 122,378 | 37,000 | 1,948 | 3,826.32 | 4.70 | 0.97 |
|  | 2 | 105,524 | 37,000 | 1,374 | 2,361.24 | 4.61 | 0.98 |
|  | 3 | 64,255 | 37,000 | 972 | 1,791.09 | 4.44 | 0.99 |
|  | 4 | 66,510 | 37,000 | 2,462 | 4,612.59 | 5.60 | 0.97 |
|  | 5 | 101,109 | 37,000 | 1,037 | 1,889.73 | 3.88 | 0.99 |
|  | 6 | 78,507 | 37,000 | 1,885 | 3,504.69 | 4.94 | 0.98 |
|  | 7 | 146,159 | 37,000 | 881 | 1,536.01 | 3.59 | 0.99 |
|  | 8 | 58,282 | 37,000 | 333 | 594.00 | 2.86 | 0.99 |
|  | 9 | 189,165 | 37,000 | 443 | 758.88 | 2.95 | 0.99 |
|  | 10 | 89,378 | 37,000 | 857 | 1,655.47 | 3.80 | 0.99 |
|  | 11 | 106,410 | 37,000 | 914 | 1,906.18 | 3.07 | 0.99 |
|  | 12 | 73,901 | 37,000 | 482 | 924.40 | 2.85 | 0.99 |
|  | 13 | 146,336 | 37,000 | 995 | 1,855.71 | 4.16 | 0.99 |
|  | 14 | 57,143 | 37,000 | 922 | 1,536.88 | 4.65 | 0.99 |
|  | 15 | 127,098 | 37,000 | 1,320 | 2,567.30 | 4.24 | 0.98 |
|  | 16 | 98,272 | 37,000 | 976 | 1,864.83 | 4.19 | 0.99 |
|  | 17 | 216,758 | 37,000 | 171 | 293.83 | 0.84 | 0.99 |
|  | 18 | 91,621 | 37,000 | 1,962 | 3,550.66 | 5.23 | 0.98 |
|  | 20 | 69,129 | 37,000 | 1,546 | 2,761.05 | 4.85 | 0.98 |

**Table S3.** Significantly different KEGG pathways of microbiota between normal skin and diabetic foot wound (DFW) tissue.

| **KEGG Orthology** | **Mean proportion (± Standard Deviations)** | | **p values** |
| --- | --- | --- | --- |
|  | **Skin** | **Tissue** |  |
| Bisphenol degradation | 0.0814 ± 0.0264 | 0.0343 ± 0.0351 | 4.90E-05 |
| Non-homologous end-joining | 0.0232 ± 0.0156 | 0.0049 ± 0.0095 | 0.0001697 |
| Caffeine metabolism | 0.0020 ± 0.0018 | 0.0000 ± 0.0000 | 0.000211 |
| Steroid biosynthesis | 0.0076 ± 0.0068 | 0.0002 ± 0.0008 | 0.0002379 |
| Butanoate metabolism | 0.8706 ± 0.1580 | 0.6559 ± 0.1829 | 0.0004864 |
| Synthesis and degradation of ketone bodies | 0.1320 ± 0.0499 | 0.0655 ± 0.0561 | 0.0004994 |
| Aminobenzoate degradation | 0.3203 ± 0.0992 | 0.1942 ± 0.1023 | 0.0005141 |
| Glycosphingolipid biosynthesis - lacto and neolacto series | 0.0001 ± 0.0001 | 0.0000 ± 0.0000 | 0.0005848 |
| Valine, leucine and isoleucine biosynthesis | 0.7180 ± 0.0970 | 0.5532 ± 0.1672 | 0.0008608 |
| Limonene and pinene degradation | 0.3067 ± 0.1137 | 0.1697 ± 0.1183 | 0.0009515 |
| Naphthalene degradation | 0.2584 ± 0.0577 | 0.1684 ± 0.0924 | 0.0011291 |
| Amyotrophic lateral sclerosis (ALS) | 0.0469 ± 0.0220 | 0.0239 ± 0.0182 | 0.0014441 |
| Epithelial cell signaling in Helicobacter pylori infection | 0.0590 ± 0.0120 | 0.0861 ± 0.0309 | 0.0015883 |
| General function prediction only_Unclassified | 3.5396 ± 0.1069 | 3.7333 ± 0.2165 | 0.0016635 |
| Chloroalkane and chloroalkene degradation | 0.2302 ± 0.0503 | 0.1558 ± 0.0804 | 0.0018436 |
| Chlorocyclohexane and chlorobenzene degradation | 0.0542 ± 0.0291 | 0.0221 ± 0.0296 | 0.0020005 |
| Valine, leucine and isoleucine degradation | 0.6826 ± 0.2272 | 0.4174 ± 0.2577 | 0.0020071 |
| PPAR signaling pathway | 0.1670 ± 0.0467 | 0.1104 ± 0.0605 | 0.0029276 |
| Drug metabolism - cytochrome P450 | 0.1386 ± 0.0660 | 0.0679 ± 0.0695 | 0.0030171 |
| Peroxisome | 0.2505 ± 0.0451 | 0.1903 ± 0.0677 | 0.0030616 |
| Metabolism of xenobiotics by cytochrome P450 | 0.1332 ± 0.0625 | 0.0666 ± 0.0665 | 0.0033267 |
| Fatty acid metabolism | 0.5859 ± 0.1838 | 0.3804 ± 0.2233 | 0.004094 |
| Caprolactam degradation | 0.1622 ± 0.0879 | 0.0703 ± 0.0978 | 0.0047197 |
| Lipopolysaccharide biosynthesis proteins | 0.2519 ± 0.1002 | 0.4700 ± 0.2883 | 0.0048613 |
| Retinol metabolism | 0.0838 ± 0.0243 | 0.0539 ± 0.0358 | 0.0052079 |
| p53 signaling pathway | 0.0102 ± 0.0137 | 0.000006 ± 0.000002 | 0.0055286 |
| Benzoate degradation | 0.4321 ± 0.1119 | 0.2926 ± 0.1717 | 0.0059057 |
| Influenza A | 0.0101 ± 0.0137 | 0.000006 ± 0.000002 | 0.0059312 |
| Toxoplasmosis | 0.0101 ± 0.0137 | 0.000006 ± 0.000002 | 0.0059312 |
| Apoptosis | 0.0172 ± 0.0190 | 0.0027 ± 0.0081 | 0.0062968 |
| Linoleic acid metabolism | 0.0432 ± 0.0127 | 0.0242 ± 0.0249 | 0.0063196 |
| Propanoate metabolism | 0.8458 ± 0.1684 | 0.6520 ± 0.2348 | 0.006508 |
| Tryptophan metabolism | 0.4645 ± 0.1550 | 0.2913 ± 0.2071 | 0.0065739 |
| Photosynthesis proteins | 0.3895 ± 0.0888 | 0.2964 ± 0.1102 | 0.0074281 |
| Vibrio cholerae pathogenic cycle | 0.0601 ± 0.0156 | 0.0873 ± 0.0379 | 0.007835 |
| Glycosyltransferases | 0.3819 ± 0.0261 | 0.4302 ± 0.0679 | 0.008005 |
| Indole alkaloid biosynthesis | 0.0001 ± 0.0001 | 0.0000 ± 0.0000 | 0.0083199 |
| RIG-I-like receptor signaling pathway | 0.0091 ± 0.0047 | 0.0031 ± 0.0081 | 0.009143 |
| Lysine degradation | 0.3923 ± 0.1325 | 0.2613 ± 0.1570 | 0.0092516 |
| Basal transcription factors | 0.0105 ± 0.0071 | 0.0032 ± 0.0089 | 0.0094035 |
| Renin-angiotensin system | 0.0014 ± 0.0015 | 0.0002 ± 0.0009 | 0.0096696 |
| Stilbenoid, diarylheptanoid and gingerol biosynthesis | 0.0169 ± 0.0130 | 0.0066 ± 0.0095 | 0.0102483 |
| Geraniol degradation | 0.2438 ± 0.1187 | 0.1290 ± 0.1436 | 0.0116772 |
| Atrazine degradation | 0.0660 ± 0.0239 | 0.0363 ± 0.0423 | 0.0128399 |
| Peptidases | 1.6047 ± 0.1561 | 1.8531 ± 0.3923 | 0.0170985 |
| Polycyclic aromatic hydrocarbon degradation | 0.1415 ± 0.0253 | 0.1085 ± 0.0505 | 0.0171752 |
| Membrane and intracellular structural molecules_Unclassified | 0.4791 ± 0.1489 | 0.6785 ± 0.3086 | 0.0176223 |
| Bile secretion | 0.0013 ± 0.0016 | 0.0002 ± 0.0007 | 0.01897 |
| Toluene degradation | 0.1658 ± 0.0331 | 0.1362 ± 0.0401 | 0.0190311 |
| Lipopolysaccharide biosynthesis | 0.1651 ± 0.0865 | 0.3161 ± 0.2470 | 0.0193831 |
| Betalain biosynthesis | 0.0002 ± 0.0002 | 0.0000005 ± 0.0000002 | 0.020365 |
| Photosynthesis | 0.3604 ± 0.0744 | 0.2858 ± 0.1097 | 0.0205382 |
| Translation proteins_Unclassified | 0.8263 ± 0.1076 | 0.9389 ± 0.1691 | 0.0208007 |
| Tyrosine metabolism | 0.4485 ± 0.0417 | 0.3845 ± 0.1065 | 0.0225756 |
| Transcription related proteins_Unclassified | 0.0121 ± 0.0083 | 0.0269 ± 0.0254 | 0.0243441 |
| NOD-like receptor signaling pathway | 0.0156 ± 0.0080 | 0.0304 ± 0.0258 | 0.0256878 |
| Inositol phosphate metabolism | 0.1808 ± 0.0337 | 0.1389 ± 0.0693 | 0.0257426 |
| Chagas disease (American trypanosomiasis) | 0.0140 ± 0.0068 | 0.0071 ± 0.0109 | 0.026413 |
| Antigen processing and presentation | 0.0136 ± 0.0056 | 0.0254 ± 0.0210 | 0.0271419 |
| Progesterone-mediated oocyte maturation | 0.0136 ± 0.0056 | 0.0254 ± 0.0210 | 0.0271419 |
| Fructose and mannose metabolism | 0.7087 ± 0.1389 | 0.8228 ± 0.1624 | 0.0271922 |
| Endocytosis | 0.0019 ± 0.0033 | 0.000009 ± 0.000003 | 0.0273669 |
| GnRH signaling pathway | 0.0019 ± 0.0033 | 0.000009 ± 0.000003 | 0.0273669 |
| Fc gamma R-mediated phagocytosis | 0.0019 ± 0.0033 | 0.00001 ± 0.000003 | 0.0274505 |
| Sulfur metabolism | 0.3143 ± 0.0434 | 0.2585 ± 0.0959 | 0.0295088 |
| Hematopoietic cell lineage | 0.0001 ± 0.0001 | 0.0000 ± 0.0000 | 0.0310998 |
| Protein digestion and absorption | 0.0100 ± 0.0089 | 0.0306 ± 0.0379 | 0.0312564 |
| Cytochrome P450 | 0.0008 ± 0.0015 | 0.0000004 ± 0.0000002 | 0.0321312 |
| Zeatin biosynthesis | 0.0457 ± 0.0114 | 0.0632 ± 0.0316 | 0.0327244 |
| C5-Branched dibasic acid metabolism | 0.3041 ± 0.0604 | 0.2348 ± 0.1208 | 0.0341067 |
| Glycan biosynthesis and metabolism_Unclassified | 0.0263 ± 0.0175 | 0.0508 ± 0.0451 | 0.036937 |
| Lipid biosynthesis proteins | 0.6555 ± 0.0784 | 0.5850 ± 0.1170 | 0.0380307 |
| beta-Alanine metabolism | 0.3688 ± 0.1085 | 0.2863 ± 0.1264 | 0.0396764 |
| Amino acid metabolism_Unclassified | 0.1786 ± 0.0226 | 0.2239 ± 0.0873 | 0.0398888 |
| Vasopressin-regulated water reabsorption | 0.00002 ± 0.00003 | 0.0000 ± 0.0000 | 0.0443088 |
| Glutathione metabolism | 0.3717 ± 0.0971 | 0.3114 ± 0.0791 | 0.0465663 |
| Glycosaminoglycan degradation | 0.0265 ± 0.0164 | 0.0627 ± 0.0728 | 0.047078 |
| Chromosome | 1.3529 ± 0.1351 | 1.4909 ± 0.2550 | 0.0471225 |
| Chaperones and folding catalysts | 0.8855 ± 0.1103 | 1.0163 ± 0.2500 | 0.047654 |
| Lipid metabolism_Unclassified | 0.1056 ± 0.0192 | 0.1293 ± 0.0460 | 0.049284 |

**Table S4.** Significantly different KEGG pathways of microbiota in diabetic foot wound (DFW) tissue between group with ESRD and group without ESRD.

| **KEGG Orthology** | **Mean proportion (± Standard Deviations)** | | **p values** |
| --- | --- | --- | --- |
|  | **ESRD Yes** | **ESRD No** |  |
| Cell division_Unclassified | 0.0464 ± 0.0233 | 0.0801 ± 0.0331 | 0.021177888 |
| Nicotinate and nicotinamide metabolism | 0.3742 ± 0.0791 | 0.5248 ± 0.1700 | 0.025469616 |
| Others_Unclassified | 1.1186 ± 0.1309 | 0.9216 ± 0.2099 | 0.026217903 |
| Riboflavin metabolism | 0.2772 ± 0.0566 | 0.3368 ± 0.0524 | 0.035204559 |
| Adipocytokine signaling pathway | 0.0491 ± 0.0340 | 0.0960 ± 0.0540 | 0.037947677 |
| Prenyltransferases | 0.3427 ± 0.0961 | 0.4452 ± 0.1061 | 0.046042668 |

**Table S5.** Significantly different KEGG pathway of microbiota in diabetic foot wound (DFW) tissue between HbA1c < 8% and > 8% groups**.**

| **KEGG Orthology** | **Mean proportion (± standard deviations)** | | **p values** |
| --- | --- | --- | --- |
|  | **HbA1c < 8%** | **HbA1c > 8%** |  |
| Sulfur relay system | 0.3745 ± 0.0739 | 0.2228 ± 0.0596 | 0.003547435 |
| Glutathione metabolism | 0.3207 ± 0.0456 | 0.2537 ± 0.0175 | 0.003572163 |
| Peptidoglycan biosynthesis | 0.8289 ± 0.1598 | 1.1093 ± 0.1311 | 0.009446527 |
| Cellular antigens | 0.0365 ± 0.0302 | 0.0946 ± 0.0316 | 0.016818375 |
| DNA replication proteins | 1.1285 ± 0.1936 | 1.4822 ± 0.2019 | 0.020691618 |
| Epithelial cell signaling in Helicobacter pylori infection | 0.0730 ± 0.0291 | 0.1129 ± 0.0211 | 0.021405764 |
| Phenylalanine metabolism | 0.2156 ± 0.1008 | 0.1035 ± 0.0466 | 0.021900708 |
| Chromosome | 1.3788 ± 0.1761 | 1.7162 ± 0.2001 | 0.023640234 |
| Ribosome | 2.3505 ± 0.5352 | 3.1467 ± 0.4555 | 0.024307633 |
| Zeatin biosynthesis | 0.0497 ± 0.0195 | 0.0918 ± 0.0254 | 0.025037796 |
| Chlorocyclohexane and chlorobenzene degradation | 0.0272 ± 0.0249 | 0.0030 ± 0.0025 | 0.025273167 |
| Function unknown_Unclassified | 1.6275 ± 0.3699 | 1.2386 ± 0.1685 | 0.028033248 |
| Cell cycle - Caulobacter | 0.4474 ± 0.1060 | 0.6178 ± 0.1050 | 0.029402386 |
| Pyrimidine metabolism | 1.7666 ± 0.3670 | 2.3202 ± 0.3368 | 0.029479417 |
| Biosynthesis of unsaturated fatty acids | 0.1545 ± 0.0652 | 0.0912 ± 0.0214 | 0.030378694 |
| DNA replication | 0.6762 ± 0.1243 | 0.8287 ± 0.0861 | 0.030987844 |
| Inositol phosphate metabolism | 0.1686 ± 0.0516 | 0.0780 ± 0.0589 | 0.034110208 |
| alpha-Linolenic acid metabolism | 0.0339 ± 0.0255 | 0.0070 ± 0.0136 | 0.034519344 |
| Arachidonic acid metabolism | 0.0608 ± 0.0356 | 0.1168 ± 0.0378 | 0.040466356 |
| Geraniol degradation | 0.1276 ± 0.0801 | 0.0580 ± 0.0121 | 0.040559599 |
| Translation factors | 0.5060 ± 0.1138 | 0.7107 ± 0.1407 | 0.041015301 |
| Metabolism of cofactors and vitamins_Unclassified | 0.1845 ± 0.0282 | 0.1353 ± 0.0340 | 0.04227954 |
| Caprolactam degradation | 0.0806 ± 0.0827 | 0.0101 ± 0.0055 | 0.042625904 |
| Terpenoid backbone biosynthesis | 0.5973 ± 0.1456 | 0.7416 ± 0.0782 | 0.045539862 |
| Purine metabolism | 2.3384 ± 0.3243 | 2.7308 ± 0.2581 | 0.046919395 |
| Drug metabolism - other enzymes | 0.3227 ± 0.0915 | 0.4560 ± 0.0931 | 0.046976759 |
| Folate biosynthesis | 0.4982 ± 0.0988 | 0.5882 ± 0.0417 | 0.047460608 |
| Mismatch repair | 0.7996 ± 0.1591 | 1.0139 ± 0.1498 | 0.04976047 |


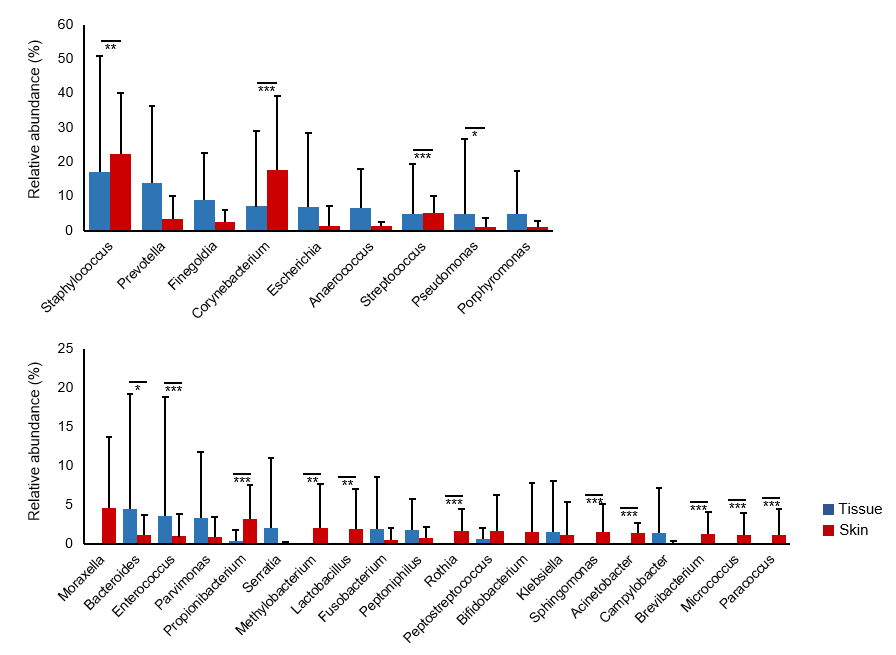


**Figure S1. Comparison of microbiota between normal foot skin and diabetic foot wound (DFW) tissue at genus level.** Frequently detected genera (> 1% of total microbiota in each sample type) were selected and compared. *p < 0.05, **p < 0.01, ***p < 0.001.


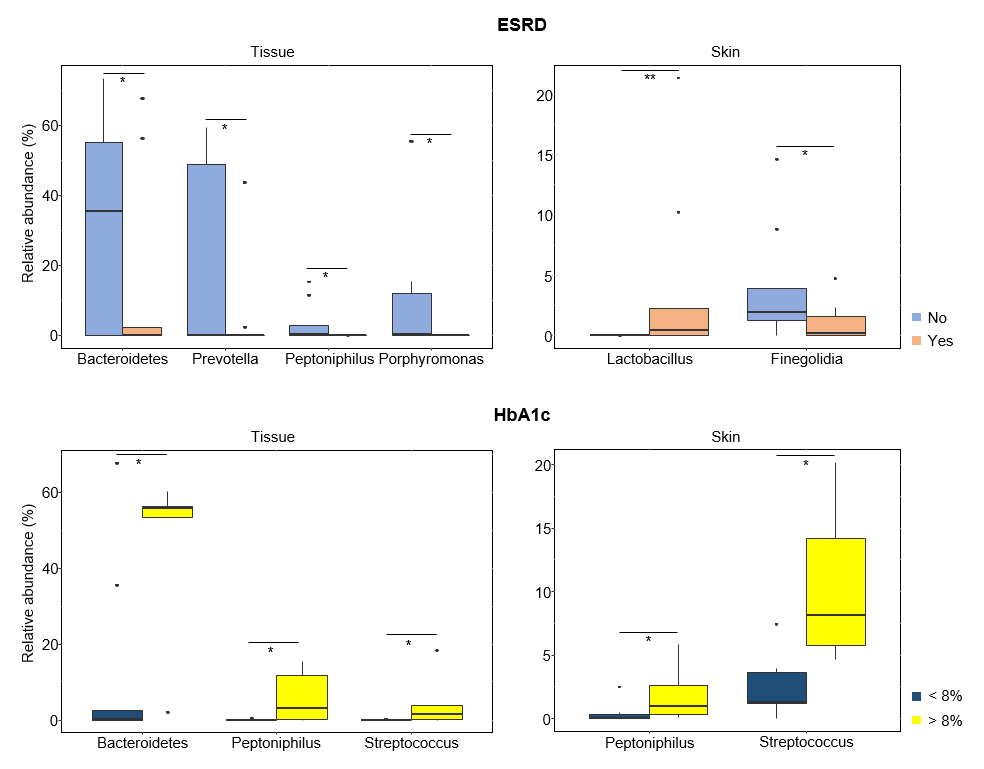


**Figure S2. Significantly different microbial taxa in skin and tissue samples were compared between clinical groups of ESRD and HbA1c.** *p < 0.05, **p < 0.01.


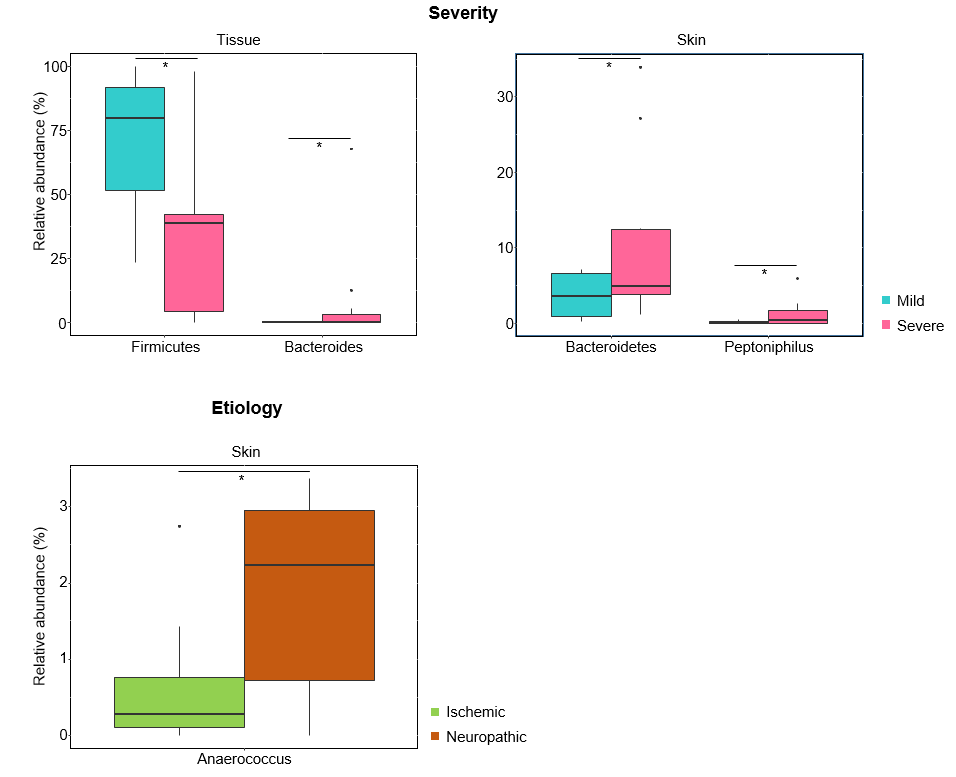


**Figure S3. Significantly different microbial taxa in skin and tissue samples were compared between clinical groups of different severity and etiology.** *p < 0.05.


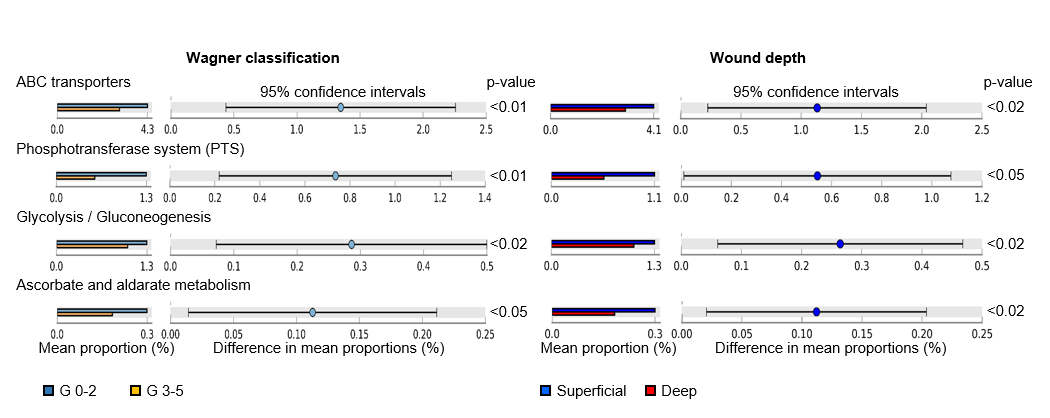


**Figure S4. Predicted functions of microbiota in DFW tissue according to Wagner classification and wound depth were compared in post-hot plots.** Four pathways were predicted to be commonly highly represented in lower grade (G0-2) of Wagner classification and superficial infection.
